# Altered resting-state network effects in patients after stroke following cortico-cortical target transcranial magnetic stimulation

**DOI:** 10.1093/braincomms/fcag305

**Published:** 2026-08-18

**Authors:** Xiang-Xin Xing, Jia-Jia Wu, Jie Ma, Mou-Xiong Zheng, Xu-Yun Hua, Jian-Guang Xu, Yong-Hui Wang

**Affiliations:** Rehabilitation Center, Qilu Hospital of Shandong University, Jinan 250012, China; Department of Rehabilitation Medicine, Yueyang Hospital, Shanghai University of Traditional Chinese Medicine, Shanghai 200437, China; Engineering Research Center of Traditional Chinese Medicine Intelligent Rehabilitation, Ministry of Education, Shanghai 201203, China; Department of Rehabilitation Medicine, Yueyang Hospital, Shanghai University of Traditional Chinese Medicine, Shanghai 200437, China; Engineering Research Center of Traditional Chinese Medicine Intelligent Rehabilitation, Ministry of Education, Shanghai 201203, China; Engineering Research Center of Traditional Chinese Medicine Intelligent Rehabilitation, Ministry of Education, Shanghai 201203, China; Department of Traumatology and Orthopedics, Shuguang Hospital of Integrated Traditional Chinese and Western Medicine, Shanghai University of Traditional Chinese Medicine, Shanghai 200000, China; Engineering Research Center of Traditional Chinese Medicine Intelligent Rehabilitation, Ministry of Education, Shanghai 201203, China; Department of Traumatology and Orthopedics, Shuguang Hospital of Integrated Traditional Chinese and Western Medicine, Shanghai University of Traditional Chinese Medicine, Shanghai 200000, China; Engineering Research Center of Traditional Chinese Medicine Intelligent Rehabilitation, Ministry of Education, Shanghai 201203, China; School of Rehabilitation Science, Shanghai University of Traditional Chinese Medicine, Shanghai 201203, China; Rehabilitation Center, Qilu Hospital of Shandong University, Jinan 250012, China

**Keywords:** cortico-cortical paired associated stimulation, resting-state network, stroke, independent component analysis, functional connectivity

## Abstract

The brain operates through large-scale, distributed networks, which are disrupted after stroke. Cortico-cortical paired associative stimulation (ccPAS) is a novel neuromodulation technique designed to induce spike-timing-dependent plasticity in specific pathways. However, its effects on large-scale resting-state networks (RSNs) in stroke patients remain unclear. In this randomized, double-blind, sham-controlled study, 31 chronic stroke patients (mean age: 49.4 ± 11.8 years) with unilateral supratentorial lesions and upper limb motor dysfunction were recruited. Participants received a 12-day intervention of either active ccPAS (*n* = 16) or sham stimulation (*n* = 15) targeting the supplementary motor area (SMA) and primary motor cortex (M1) with a +6 ms interval, and routine rehabilitation. Resting-state functional MRI was acquired pre- and post-intervention. Independent component analysis was used to identify RSNs, and functional network connectivity (FNC) was assessed. Compared to the sham group, the ccPAS group exhibited significantly integrated functional connectivity within three visual networks, the bilateral frontoparietal network, dorsal attention network, default mode network, the sensorimotor network and auditory network (*P* < 0.001). FNC analysis revealed a reduced time lag between the primary visual network and the sensorimotor network post-ccPAS (*P* < 0.05). ccPAS targeting the SMA-M1 pathway induces widespread reorganization of RSNs, enhancing connectivity within and between networks subserving attention, visuospatial processing and sensorimotor integration. This pattern of change is consistent with mechanisms of adaptive neuroplasticity and suggests that ccPAS can facilitate recovery by modulating higher-order, distributed brain networks in chronic stroke patients. Chinese Clinical Trial Registry: ChiCTR2000036685.

## Teaser

Xing *et al*. demonstrated that cortico-cortical paired associative stimulation targeting the primary motor cortex and supplementary motor area reorganized large-scale brain network connectivity in stroke patients. Their randomized trial showed the stimulation enhanced integration within and between key networks, suggesting it promotes recovery by facilitating adaptive plasticity across functional systems.

## Graphical Abstract

**Graphical Abstract:**
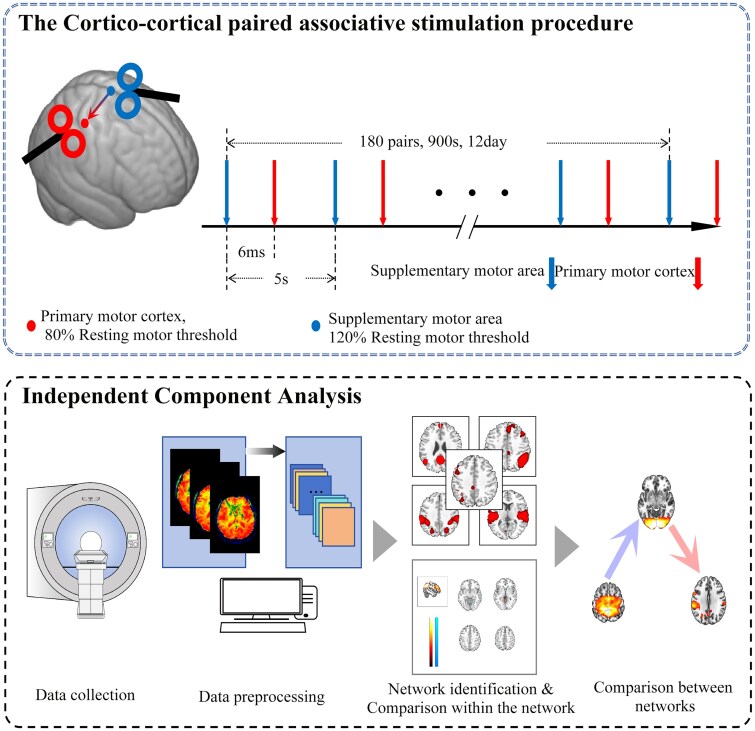

## Introduction

The human brain operates through dynamic interactions among specialized neural networks, where localized regions coordinate by long-range connections to support complex behaviours.^1^ After a stroke event, this intricate balance is disrupted, not only at the lesion region but also across distributed networks, a phenomenon termed ‘network diaschisis’.^2^ For instance, focal damage to subcortical motor pathways can decouple the supplementary motor area (SMA) from frontoparietal circuits, impairing movement planning.^3^ While spontaneous neuroplasticity partially restores connectivity, residual deficits in motor control often persist, particularly in chronic stages.^4^ Therefore, understanding how to amplify adaptive network reorganization has thus emerged as a pivotal challenge in neurorehabilitation.

Transcranial magnetic stimulation (TMS), a non-invasive neuromodulation tool, has offered unique potential to probe and reshape brain networks with frequency-dependent protocols. Currently widely recognized protocols like repetitive TMS (rTMS) modulate cortical excitability through frequency-dependent effects; high-frequency rTMS enhances activity,^5,6^ whereas low-frequency protocols suppress pathological hyperactivity.^7^ Such protocols primarily target isolated nodes, such as primary motor cortex (M1), dorsolateral prefrontal cortex, with limited capacity to address distributed network disruptions.^5^ For example, while rTMS over M1 may transiently improve hand function, it fails to restore SMA-dorsal attention network (DAN) interactions critical for goal-directed movements.^8^ Recent investigations emphasized the need for circuit-specific interventions that leverage spike-timing-dependent plasticity (STDP) to strengthen cortico-cortical pathways critical for motor recovery.^9^

Cortico-cortical paired associative stimulation (ccPAS) represents a paradigm shift through leveraging STDP to strengthen synaptic connections between predefined nodes. By delivering paired pulses to two interconnected regions with millisecond precision, ccPAS mimics the effect of Hebbian plasticity and selectively enhances synaptic connections in targeted pathways.^10^ For instance, stimulating premotor-M1 circuits with an 8 ms interstimulus interval improves grasping-related connectivity in healthy adults,^11^ while SMA-M1 paired stimulation at +6 ms interstimulus interval strengthens feedforward projections involved in movement initiation.^12^ Crucially, animal studies suggest that ccPAS effects propagate beyond stimulated sites, inducing global network reorganization through heterosynaptic mechanisms.^8^ The mechanisms by which ccPAS modulates large-scale resting-state networks (RSNs) in stroke—particularly those governing sensorimotor integration and cognitive motor control—are not clarified.

Recent investigations into post-stroke network reorganization have begun to capture more complex, high-order interactions. Studies using EEG have shown that rehabilitation can modulate directed functional connectivity within the motor network, revealing a reorganization that is not apparent from standard functional connectivity analyses alone.^13^ Furthermore, there is growing recognition that high-order dependencies—statistical interactions between multiple brain regions that cannot be explained by lower-order effects—play a crucial role in brain function and are significantly altered following stroke.^14,15^ Advanced analytical frameworks are now being used to uncover this hierarchical organization within motor networks.^16^ These findings underscore the complexity of network-level changes post-stroke and highlight the need for interventions, like ccPAS, that are designed to modulate specific pathways within these distributed, high-order networks.

Post-stroke motor dysfunction is increasingly recognized as a multi-network disorder. Resting-state fMRI studies identify three characteristic disruptions: (i) enhanced connectivity within the default mode network (DMN), reflecting maladaptive self-referential processing^2^; (ii) reduced connectivity in the DAN, impairing attention-driven motor control^17^; and (iii) aberrant sensorimotor network (SMN) integration with visual circuits, hindering error correction.^18^ Conventional rehabilitation strategies, while effective in enhancing local M1 activity, often fail to repair inter-network dynamics. For example, constraint-induced movement therapy improves hand function but does not normalize DMN–SMN decoupling.^19^ We hypothesize that dual-site ccPAS targeting SMA and M1—key hubs for movement planning and execution—can drive multimodal network plasticity, enhancing not only SMN integrity but also DAN and visual network (VN) connectivity through cross-modal compensation.

In the present study, we combined ccPAS with independent component analysis (ICA), a commonly used computational method of fMRI for calculating group differences between network, in an attempt to explore a network-targeted neuromodulation framework. This approach not only promotes mechanistic understanding of ccPAS but also informs personalised rehabilitation strategies that target individual network characteristics.

## Materials and methods

### Participants

The participants were chronic stroke patients who were admitted in Yueyang Hospital of Integrated Traditional Chinese and Western Medicine Affiliated to Shanghai University of TCM. Inclusion criteria for all participants were as follows: (i) Patients diagnosed with stroke via clinical evaluation (Chinese Guidelines for the Diagnosis and Treatment of Acute Ischemic Stroke 2018)^20^; (ii) Age ≥18 and ≤70 years, no gender limitation; (iii) Course of disease ≥3 and ≤12 months; (iv) Unilateral supratentorial lesion confirmed by MRI or CT, caused by either ischaemic stroke or intracerebral haemorrhage, and the lesion was not located in the regions of M1 and SMA; (v) education above primary school, normal cognitive function (minimum mental state examination (MMSE) corresponding to critical value above different education level). Exclusion criteria were as follows: (i) Serious heart, liver and kidney diseases or infectious diseases; (ii) a history of epilepsy, psychiatric disorders or other diseases with severe cognitive or emotional dysfunction; (iii) MRI and TMS contraindication implants in the body of participants; (iv) Recent use of drugs for prevention or anti-epilepsy and cognitive improvement; (v) Pregnant or lactating women.

The study was approved by the ethics committee (No. KYSKSB2020-116) and informed consent has been signed by patients or their families (Chinese Clinical Trial Registry: ChiCTR2000036685).

### Evaluation of motor cortex excitability

Before interventions, the excitability of M1 in the contralesional hemisphere was assessed with motor evoked potential (MEP) by monopulse TMS. MagTD magnetic field stimulator from Wuhan IRUIDE Group Co., LTD (Wuhan, China) was utilized. All the assessments were completed by the same doctor, and the assessment time was basically consistent among different participants. The participants were asked to remain awake during the evaluation.

For the motor representative region of the first dorsolateral interosseous muscle in the healthy side hand, the largest stable MEP was continuously extracted, namely, M1 region. MEPs were recorded using surface electrodes positioned in a belly-tendon montage. Resting motor threshold (RMT) was defined as the minimum output intensity, eliciting at least 5 out of 10 MEPs higher than 100 µV.^21^

### Study design and interventions

After providing informed consent, all participants completed a baseline assessment (T0) comprising fMRI scanning, RMT measurement and clinical evaluation. Following T0, participants were randomly allocated to either the ccPAS or sham group. The intervention phase consisted of 12 daily sessions. Each daily session included 15 min of ccPAS or sham stimulation. A post-intervention assessment (T1), identical to T0, was conducted after the final intervention session. Routine rehabilitation services mainly consisted of daily physical therapy and occupational therapy by the same experienced therapist.

### ccPAS

During the ccPAS approach, the participant was asked to lie comfortably in a recliner, with both forearms on a pillow placed on his/her legs in advance. His/her head was resting on the back of the chair for support, maintaining a completely relaxed posture.

Parameters of ccPAS are as follows:

#### Stimulation intensity

The maximum surface magnetic field strength of the equipment was 6 T. The motor threshold and MEP were monitored by monopulse stimulation on the M1 of the contralesional hemisphere, with the reference site being the motor representative region of the first dorsolateral interosseous muscle of the unaffected upper extremity. The intensity was recorded with silver–silver chloride electrode of the surface electromyogram. RMT of the M1 region in the contralesional hemisphere was used as reference for comparison.^22^ The stimulation intensity at M1 was set as 80% RMT. The coil was tangent to the scalp, and the midline was rotated 45°, so that the induced current in M1 was conducted from back to middle to forward, which was considered the position of the best therapeutic effect. The stimulation intensity at SMA was set at 120% RMT, and the induced current flowed along the front and back direction.^23^

#### Stimulation sites

Before intervention, a positioning cap (Wuhan YIRUIDE Group Co., LTD) of the standard 10–20 EEG system was worn by the participant to locate SMA and M1. One 8-shaped coil was placed at the M1 of the lesioned hemisphere, symmetrically to the site in the contralesional hemisphere where MEP was elicited when the stimulus intensity was measured. Another 8-shaped coil was placed at SMA of the lesioned hemisphere. The position of the SMA placed 3 cm in front of the central zero (Cz).

#### Frequency, number of stimulation and others

According to Arai *et al*.’s study, the time interval between SMA and M1 regions was determined as +6 ms.^23^ The once-daily ccPAS stimulation regimen consisted of 180 paired TMS pulses at a frequency of 0.2 Hz, and each process lasted 15 min.

### Sham stimulation

The two coils were placed vertically to the scalp of participants during sham stimulations. The stimulation parameters in the sham group were the same as the ccPAS group. The sound produced by the coil during stimulation had virtually no effect on the cerebral cortex.

### fMRI data analysis

fMRI images of brain were collected from all participants pre-intervention and on the 14th day after cease of intervention. A 3.0 T tandem uMR 770 magnetic resonance scanning imaging system (United Imaging, Shanghai, China) was used with standard cranial 32-channel coils. To avoid diversity of brain activity at different times of the day, scanning was performed between 16:30 and 19:30 in participants. During scanning, the participant lied in supine position, with eyes closed. Ear plugs were worn to reduce noise interference, and the head was fixed in the coil. Patients were instructed to stay awake, breathe calmly, and lie still as much as possible.

### Scanning parameters

All patients underwent resting fMRI (EPI_BOLD) covering the whole brain. Resting-state fMRI were collected with plane echo sequence (EPI_BOLD). The scanning parameters were as follows: slice number = 43; matrix size = 64 × 64; FOV = 240 × 240 mm^2^; TR/TE = 3000/30 ms; flip angle = 90°; slice thickness = 3.0 mm; voxel size = 3.2 × 3.2 × 3.40 mm^3^; number of repetitions = 240 for a total acquisition time of 12 min.

### Preprocessing

Data preprocessing procedures were performed using the Statistical Parametric Mapping 12 (SPM 12) toolbox (http://www.fil.ion.ucl.ac.uk/spm/) based on the MATLAB 2013b platform (The Mathworks, Inc., Natick; The Statistical parameters of US). The first 10 volumes were removed to eliminate unstable signals. In participants with left hemisphere affected, brains were left-to-right flipped so that the affected side of the brain was localized to one side of the hemisphere.^21^ Subsequent preprocessing steps included slice timing, head motion correction (corrected by realigning all functional images to the first volume using a six-parameter rigid-body transformation), coregistration to individual anatomical images, spatial normalization to the EPI template of the Montreal Neurological Institute (MNI) space, resampling to 3.0 × 3.0 × 3.0 mm^3^ and smoothing with a 6-mm full-width at half-maximum Gaussian kernel. Linear detrending and bandpass filtering (0.01–0.08 Hz) were further carried out. Finally, the nuisance signals, including the averaged signal from white matter, cerebrospinal fluid and Friston 24 head motion parameters (the six motion parameters, their first-order temporal derivatives and their squares), were regressed out. Subjects with excessive head motion (maximum translation > 2.0 mm or maximum rotation > 2.0°) were excluded from further analysis. After preprocessing, the data of 15 ccPAS group subjects (14 male) and 16 sham group subjects (14 male) passed the head movement correction and image screening.

### Analysis of intrinsic brain functional connectivity in RSNs

The GIFT software (https://www.nitrc.org/projects/gift, version 4.0a) was used for analysing the independent components (ICs). Dimension reduction, ICA informax algorithm and back reconstruction were sequentially preformed in an individual brain. The brain of each participant was decomposed into 40 ICs and group‑information‑guided independent component analysis (GIG-ICA) was performed 100 times. Ten meaningful RSNs were identified by the anatomically and functionally classical templates.^24^

Each IC was thresholded using a random-effect one-sample *t*-test by a false discovery rate (*P*_FDR correction_ < 0.05) to remove atypical voxels with small correlation coefficients and retain highly correlated voxels. The mean time course was calculated by averaging the time courses within each RSN mask obtained from the ICA processing. It is important to note that while the ICA algorithm maximizes the statistical independence of the component spatial maps, the derived time courses can still exhibit meaningful temporal correlations that reflect synchronized neural activity between networks.^25,26^ Pearson’s correlation coefficients of the mean time courses between each pair of RSNs in individual participants were calculated and then Fisher’s *z* transformation was performed to make Pearson’s *r* (correlation coefficient) normally distributed. For between-group comparisons of spatial maps, these statistical maps were thresholded using a voxel-level height threshold of *P* < 0.001 and a cluster extent threshold of *k* ≥ 10 voxels. This cluster size was determined to be more conservative than the minimum cluster size required for at *P* < 0.05, as conducted with the AlphaSim-corrected method. Then, two-sample *t*-tests were used to obtain the group differences of the *z*-maps of the RSNs. The group-level comparisons were tested using general linear model analysis with age and sex as covariates.

### Analysis of functional connectivity between RSNs functional network connectivity analysis

General linear model was performed to explore between-group difference of functional connectivity (FC) between each pair of networks. The functional network connectivity (FNC) toolbox was used to examine the temporal correlation between identified networks (http://trendscenter.org/software/). The statistical significance of between-group differences in correlation coefficients was assessed at *P* < 0.05.

## Results

### Identification of RSNs

The baseline demographic and clinical characteristics of the two groups are summarized in Table 1. There were no significant differences between the groups in terms of age, gender, disease duration, stroke type, lesion side, comorbidities, cognitive status (MMSE) or cortical excitability (RMT) (all *P* > 0.05).

**Table 1:** The baseline demographic and clinical characteristics of the two groups

	ccPAS group (*n* = 15)	Sham group (*n* = 16)	*P*
Age (years)	47.60 ± 13.10	51.13 ± 10.60	0.414
Gender (male/female)	14/1	14/2	1.000
Course of disease (month)	7.40 ± 3.60	7.06 ± 3.60	0.798
Stroke type (infarction/haemorrhage)	10/5	12/4	0.908
Side of lesion (left/right)	7/8	9/7	0.862
Hypertension	13/2	10/6	0.260
Diabetes	4/11	6/10	0.795
MMSE	27.87 ± 2.48	28.00 ± 2.50	0.883
RMT	44.33 ± 9.93	48.06 ± 6.77	0.229

**Figure 1:**
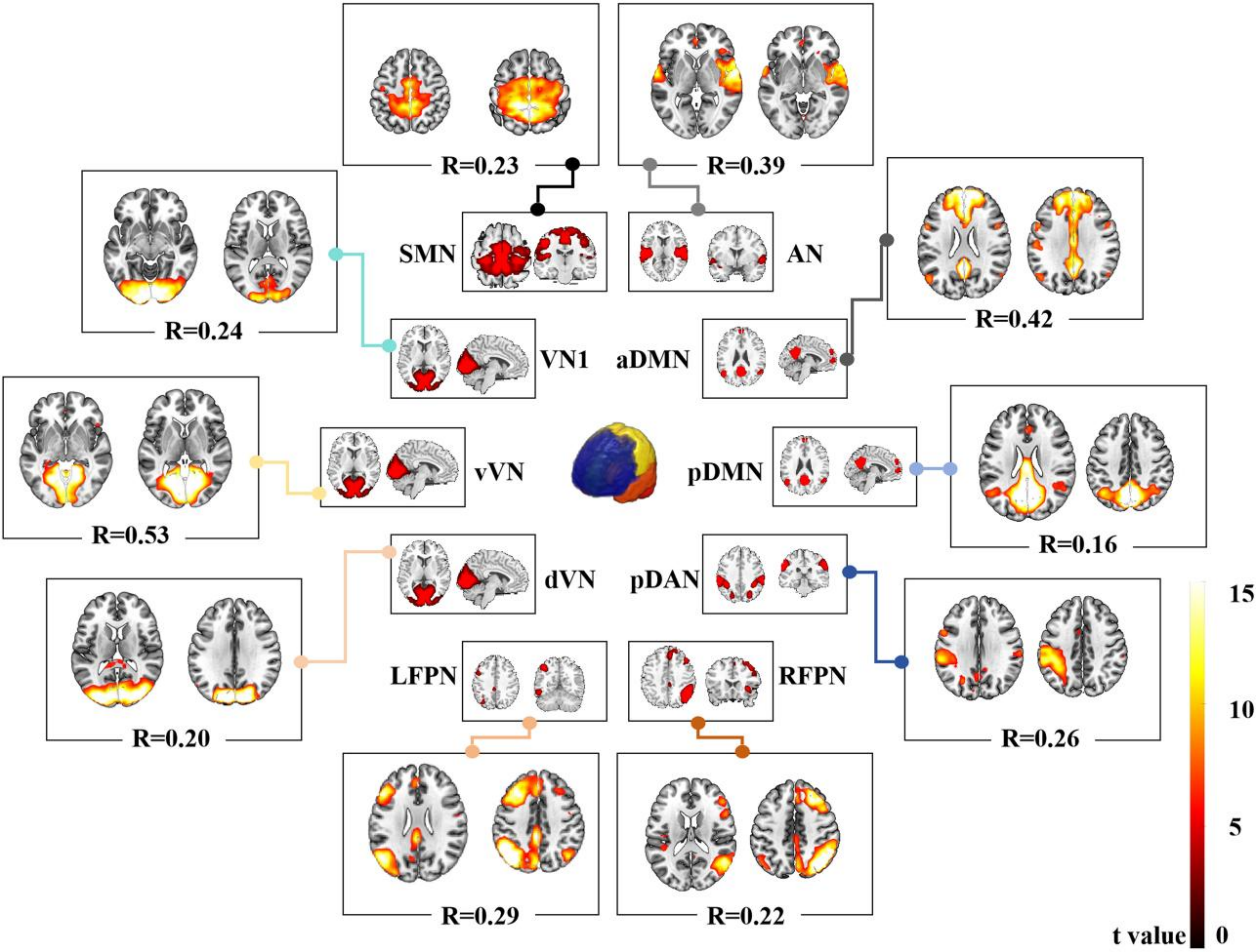
**The RSN screened by correlation analysis with the template.** Each IC was thresholded using a random-effect one-sample *t*-test by a false discovery rate (*P*_FDR correction_ < 0.05) to remove atypical voxels with small correlation coefficients and retain highly correlated voxels. Value *r* represents the correlation coefficient with the template (*N* = 31 subjects, *P* < 0.05). SMN, *t* > 2.45; aDMN, *t* > 2.22; pDMN, *t* > 2.42; DAN, *t* > 2.35; FPN, *t* > 2.31 (right), *t* > 2.26 (left); AN, *t* > 2.30; VN1, *t* > 2.48; vVN, *t* > 2.46; and dVN, *t* > 2.35.

Then, ICA was performed. After correlation analysis with the reference templates, 10 meaningful RSNs were screened: SMN, anterior DMN (aDMN), posterior DMN (pDMN), DAN, bilateral frontal parietal network (FPN), auditory network (AN), primary VN (VN1), ventral VN (vVN) and dorsal VN (dVN) (Fig. 1).

### Between-group differences within RSNs

**Figure 2:**
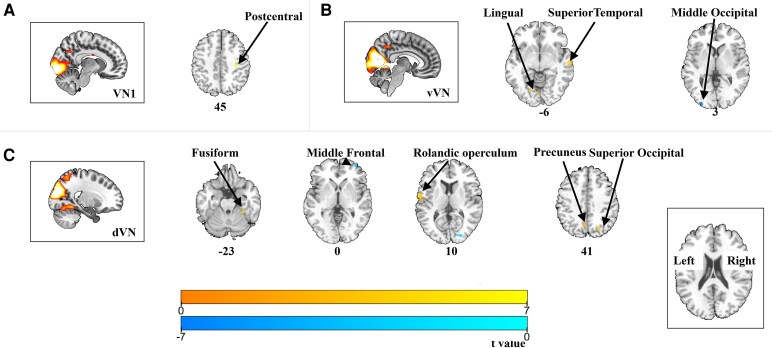
**Comparison of the differences within the VNs between the ccPAS group and the sham group.** (**A**) VN1. (**B**) vVN. (**C**) dVN. Warm colour represents increased functional connectivity value on the 14th day post-intervention compared with pre-intervention status, while cold colour represents decreased functional connectivity (*N* = 31 subjects) (two-sample *t*-test, AlphaSim-corrected, voxel-level *P* = 0.001, cluster-level *P* = 0.05; a more conservative cluster extent threshold of *k* ≥ 10 was applied, *t* = 3.23). The numbers displayed below each brain image are the MNI stereotactic coordinates for the corresponding axial slice.

The results of this study observed significant alterations in FC within these RSNs. In the VN, compared with the Sham group, the ccPAS group showed increased FC between the right anterior central gyrus of the primary VN and the left lingual gyrus and the right superior temporal gyrus of the vVN, and decreased FC of the left middle occipital gyrus. In the dVN, increased FC in the left central sulcus cover, precuneus and right fusiform gyrus, while decreased FC was found in the right middle frontal gyrus, wedge lobe and superior occipital gyrus (*P* < 0.001, uncorrected) (Fig. 2, Table 2).

**Table 2:** Altered functional connectivity within the VNs between the two groups of subjects^^a^^

RSN	Contrast name	Region label	Extent	*t*-value	MNI coordinates		
					*x*	*y*	*z*
Primary VN	ccPAS group > Sham group	Right postcentral gyrus	10	4.974	33	−24	45
Ventral VN	ccPAS group > Sham group	Right superior temporal gyrus	17	4.620	63	−15	−6
		Left lingual gyrus	23	3.969	−12	−75	−9
	ccPAS group < Sham group	Left middle occipital gyrus	15	−4.568	−36	−96	0
Dorsal VN	ccPAS group > Sham group	Left Rolandic operculum	33	5.428	−60	−3	9
		Right fusiform gyrus	13	4.701	33	−39	−24
		Left precuneus	12	3.920	−9	−60	39
	ccPAS group < Sham group	Right superior occipital gyrus	17	−4.229	24	−87	9
		Right middle frontal gyrus	11	−4.071	36	57	0

^a^AlphaSim-corrected, voxel-level *P* = 0.001, cluster-level *P* = 0.05; a more conservative cluster extent threshold of *k* ≥ 10 was applied.

**Figure 3:**
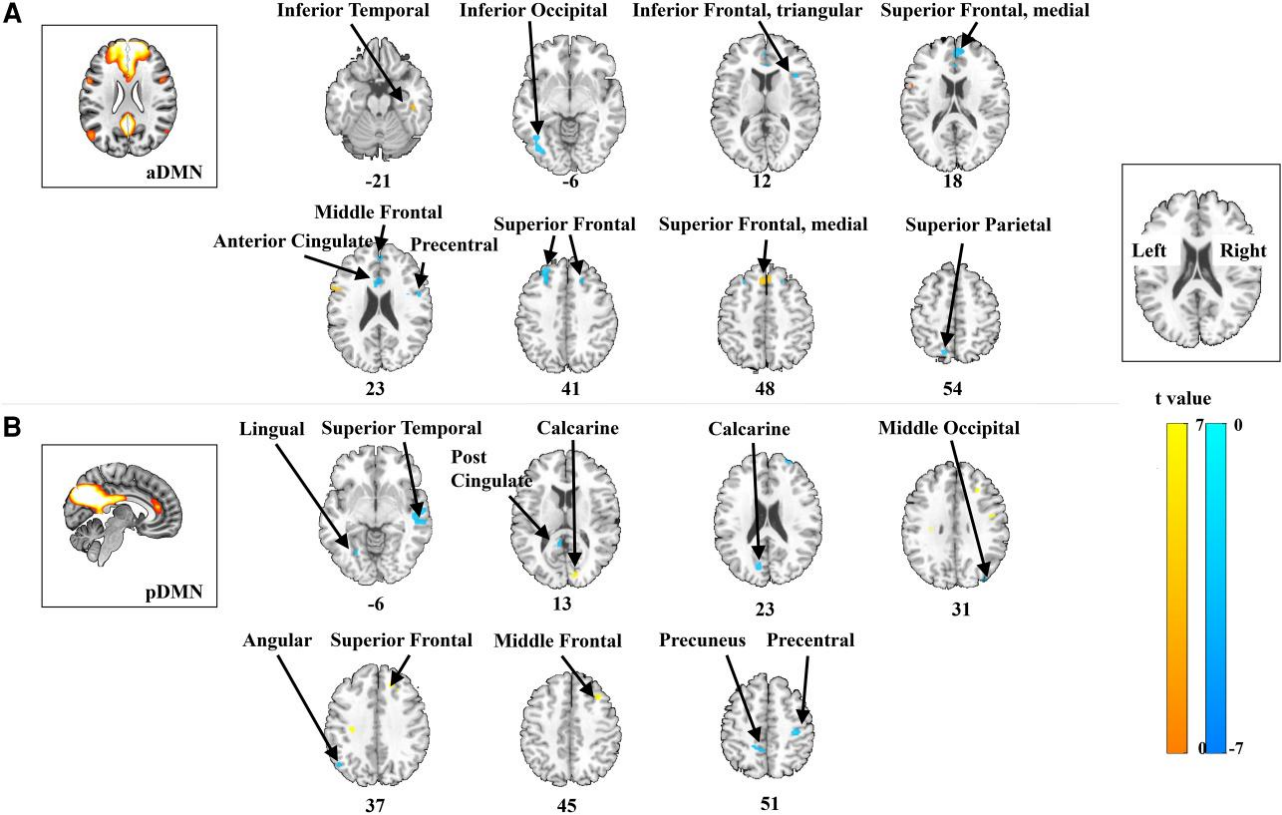
**Comparison of the DMN differences between the ccPAS group and the sham group.** (**A**) aDMN and (**B**) pDMN. Warm colour represents increased functional connectivity value on the 14th day post-intervention compared with pre-intervention status, while cold colour represents decreased functional connectivity (*N* = 31 subjects) (two-sample *t*-test, AlphaSim-corrected, voxel-level *P* = 0.001, cluster-level *P* = 0.05; a more conservative cluster extent threshold of *k* ≥ 10 was applied, *t* = 3.23). The numbers displayed below each brain image are the MNI stereotactic coordinates for the corresponding axial slice.

In the DMN, compared with the sham group, the FC of the left medial superior frontal gyrus and anterior central gyrus of the DMN before the ccPAS group increased, and the FC of the left superior frontal gyrus, anterior cingulate gyrus, superior parietal gyrus, inferior occipital gyrus, right middle frontal gyrus, superior frontal gyrus, inferior frontal gyrus of triangle and anterior central gyrus decreased significantly. In the pDMN, the FC of the right middle frontal gyrus, inferior frontal gyrus and the cortex around the spastic fissure increased, while the FC of the left lingual gyrus, posterior cingulate gyrus, angular gyrus and the cortex around the spastic fissure, the right anterior central gyrus, middle occipital gyrus and superior temporal gyrus decreased (*P* < 0.001, uncorrected) (Fig. 3, Table 3).

**Table 3:** Altered functional connectivity within the DMNs of the two groups of subjects^a^

RSN	Contrast name	Region label	Extent	*t*-value	MNI coordinates		
					*x*	*y*	*z*
Anterior DMN	ccPAS group > Sham group	Left superior frontal gyrus, medial	35	4.725	−3	30	48
		Left precentral gyrus	21	4.707	−60	9	27
		Right inferior temporal gyrus	10	4.242	45	−27	−21
	ccPAS group < Sham group	Left superior frontal gyrus, dorsolateral	70	−5.788	−27	42	39
		Right middle frontal gyrus	17	−5.198	27	36	27
		Right precentral gyrus	21	−4.924	48	3	30
		Left anterior cingulate and paracingulate gyri	23	−4.579	−6	12	27
		Left superior frontal gyrus, medial	46	−4.505	3	48	18
		Left superior parietal gyrus	11	−4.395	−15	−66	54
		Right inferior frontal gyrus, triangular part	13	−4.358	39	21	12
		Right superior frontal gyrus, dorsolateral	22	−4.268	21	63	3
		Left inferior occipital gyrus	17	−3.935	−42	−72	−6
pDMN	ccPAS group > Sham group	Right superior frontal gyrus, dorsolateral	11	4.931	21	36	39
		Right middle frontal gyrus	13	4.701	36	24	45
		Right calcarine fissure and surrounding cortex	11	4.414	12	−84	12
	ccPAS group < Sham group	Left calcarine fissure and surrounding cortex	23	−5.712	−12	−72	18
		Right precentral gyrus	21	−5.673	36	−24	51
		Right superior frontal gyrus, dorsolateral	18	−5.335	27	60	21
		Right middle occipital gyrus	10	−4.729	36	−84	30
		Left angular gyrus	10	−4.656	−48	−66	39
		Right superior temporal gyrus	57	−4.625	57	−12	−9
		Left posterior cingulate gyrus	10	−4.503	−6	−45	12
		Left lingual gyrus	11	−4.028	−24	−63	−6

^a^AlphaSim-corrected, voxel-level *P* = 0.001, cluster-level *P* = 0.05; a more conservative cluster extent threshold of *k* ≥ 10 was applied.

**Figure 4:**
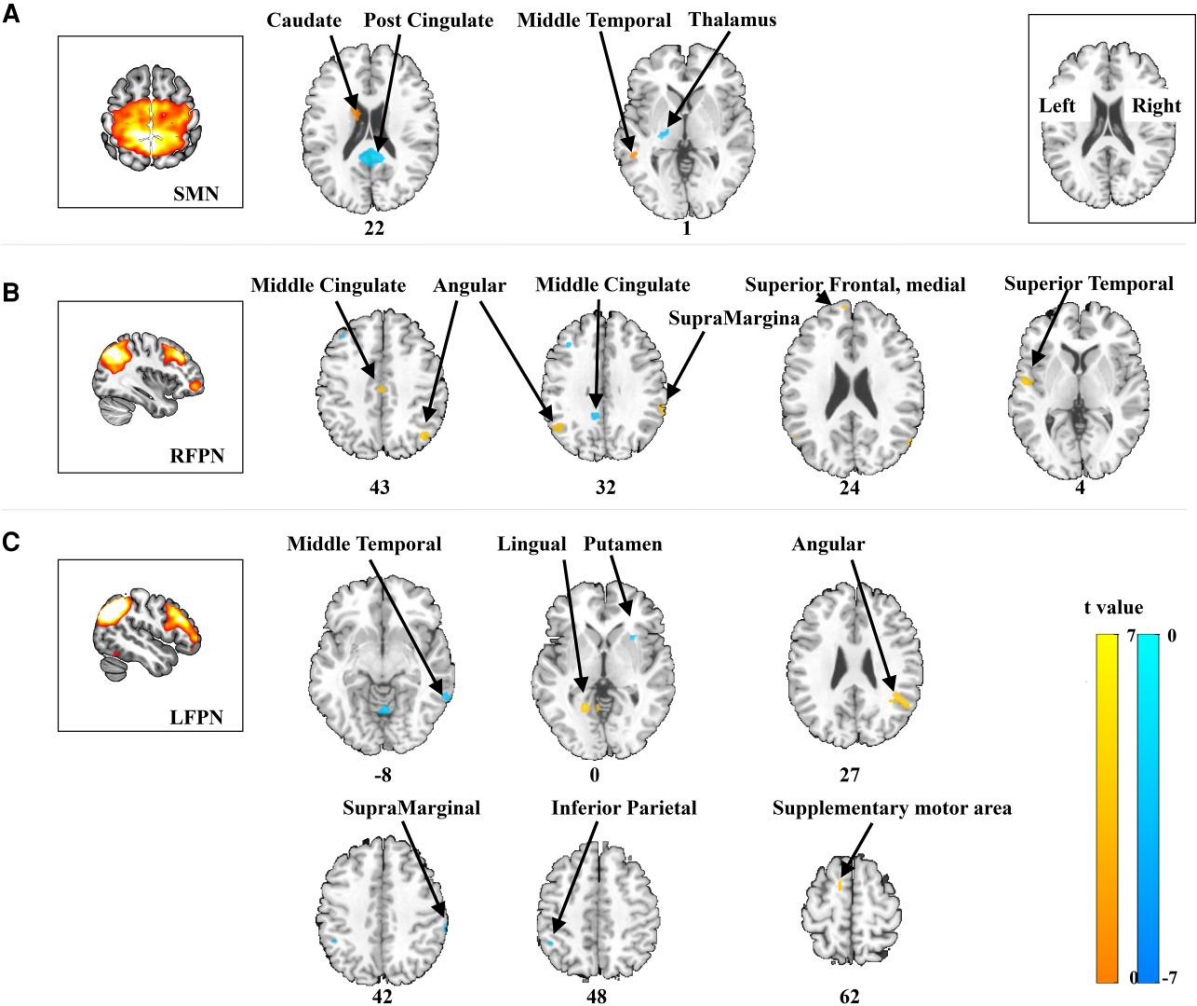
**Comparison of within-network differences in the SMN and bilateral FPNs between the ccPAS group and sham group.** (**A**) SMN, (**B**) RFPN, and (**C**) LFPN. Warm colour represents increased functional connectivity value on the 14th day post-intervention compared with pre-intervention status, while cold colour represents decreased functional connectivity (*N* = 31 subjects) (two-sample *t*-test, AlphaSim-corrected, voxel-level *P* = 0.001, cluster-level *P* = 0.05; a more conservative cluster extent threshold of *k* ≥ 10 was applied, *t* = 3.23). The numbers displayed below each brain image are the MNI stereotactic coordinates for the corresponding axial slice.

Compared with the sham group, the ccPAS group showed increased FNC in the left caudate nucleus and the right middle temporal gyrus in the SMN in the ccPAS group increased, while the FC of the right posterior cingulate gyrus and the left thalamus decreased. In the right FPN, the FC in the bilateral angular gyrus, right superior marginal gyrus, middle occipital gyrus, left superior temporal gyrus, middle cingulate gyrus and medial superior frontal lobe increased, while the FC of left middle frontal gyrus decreased. In the left FPN, the FC in the left lingual gyrus, auxiliary motor area and right angular gyrus increased, while the FC in the right putamen, middle temporal gyrus, supramarginal gyrus and left inferior parietal lobe decreased (*P* < 0.001, uncorrected) (Fig. 4, Table 4).

**Table 4:** Altered functional connectivity within the SMN, FPNs of the two groups of subjects^a^

RSN	Contrast name	Region label	Extent	*t*-value	MNI coordinates		
					*x*	*y*	*z*
SMN	ccPAS group > Sham group	Left caudate nucleus	21	5.037	−9	3	18
		Left middle temporal gyrus	13	4.340	−54	−39	0
	ccPAS group < Sham group	Right posterior cingulate gyrus	72	−6.026	6	−45	18
		Left thalamus	12	−4.055	−15	−9	6
Right FPN	ccPAS group > Sham group	Right supramarginal gyrus	32	6.116	66	−42	33
		Right angular gyrus	23	5.816	42	−66	45
		Right middle occipital gyrus	38	5.108	57	−66	24
		Left superior temporal gyrus	17	4.815	−54	−6	3
		Left angular gyrus	32	4.745	−51	−57	30
		Left median cingulate and paracingulate gyri	44	4.743	−3	−30	48
		Left superior frontal gyrus, medial	11	4.030	−6	63	24
	ccPAS group < Sham group	Left middle frontal gyrus	26	−6.260	−39	33	39
		Left median cingulate and paracingulate gyri	14	−4.150	−9	−48	33
Left FPN	ccPAS group > Sham group	Left lingual gyrus	28	5.176	−21	−54	0
		Right angular gyrus	30	5.100	51	−54	30
		Left SMA	10	4.835	−12	3	60
	ccPAS group < Sham group	Right putamen	11	−4.959	30	15	0
		Right middle temporal gyrus	12	−4.385	63	−48	−9
		Right supramarginal gyrus	15	−4.353	66	−30	42
		Left inferior parietal	13	−4.117	−48	−48	48

^a^AlphaSim-corrected, voxel-level *P* = 0.001, cluster-level *P* = 0.05; a more conservative cluster extent threshold of *k* ≥ 10 was applied.

**Figure 5:**
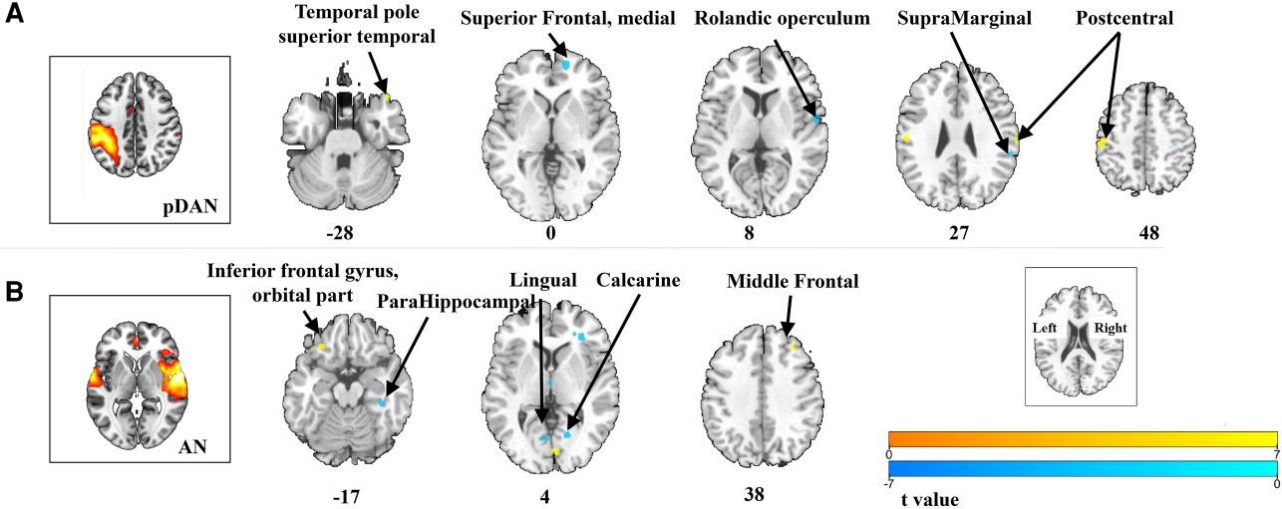
**Comparison of pDAN and AN between the ccPAS group and sham group.** (**A**) pDAN and (**B**) AN. Warm colour represents increased functional connectivity value on the 14th day post-intervention compared with pre-intervention status, while cold colour represents decreased functional connectivity (*N* = 31 subjects) (two-sample *t*-test, AlphaSim-corrected, voxel-level *P* = 0.001, cluster-level *P* = 0.05; a more conservative cluster extent threshold of *k* ≥ 10 was applied, *t* = 3.23). The numbers displayed below each brain image are the MNI stereotactic coordinates for the corresponding axial slice.

Compared with the sham group, the ccPAS group showed increased FC in the bilateral posterior central gyrus, the right temporal pole and the superior temporal gyrus in the DAN, and decreased FC in the right superior marginal gyrus, the medial superior frontal gyrus and the central sulcus cover. The FC of the left lingual gyrus, the posterior orbitofrontal lobe and the right middle frontal gyrus in the AN increased, while the FC in the right parahippocampal gyrus and the cortex around the talus fissure decreased (*P* < 0.001, uncorrected) (Fig. 5, Table 5).

**Table 5:** Altered functional connectivity within the DAN, AN of the two groups of subjects^a^

RSN	Contrast name	Region label	Extent	*t*-value	MNI coordinates		
					*x*	*y*	*z*
DAN	ccPAS group > Sham group	Left postcentral gyrus	211	5.489	−54	−24	51
		Right superior temporal gyrus, temporal pole	13	4.707	45	27	−24
		Right postcentral gyrus	16	4.603	66	−15	27
	ccPAS group < Sham group	Right supramarginal gyrus	11	−4.364	60	−33	27
		Right superior frontal gyrus, medial	14	−4.324	15	54	3
		Right Rolandic operculum	15	−4.256	57	0	9
AN	ccPAS group > Sham group	Left lingual gyrus	19	4.889	3	−81	3
		Left inferior frontal gyrus, orbital part	20	4.710	−30	33	−15
		Right middle frontal gyrus	10	4.404	33	39	39
	ccPAS group < Sham group	Right parahippocampal gyrus	21	−4.372	36	−30	−18
		Right calcarine fissure and surrounding cortex	10	−4.144	18	−66	3

^a^AlphaSim-corrected, voxel-level *P* = 0.001, cluster-level *P* = 0.05; a more conservative cluster extent threshold of *k* ≥ 10 was applied.

### Effects of ccPAS on FNC

**Figure 6:**
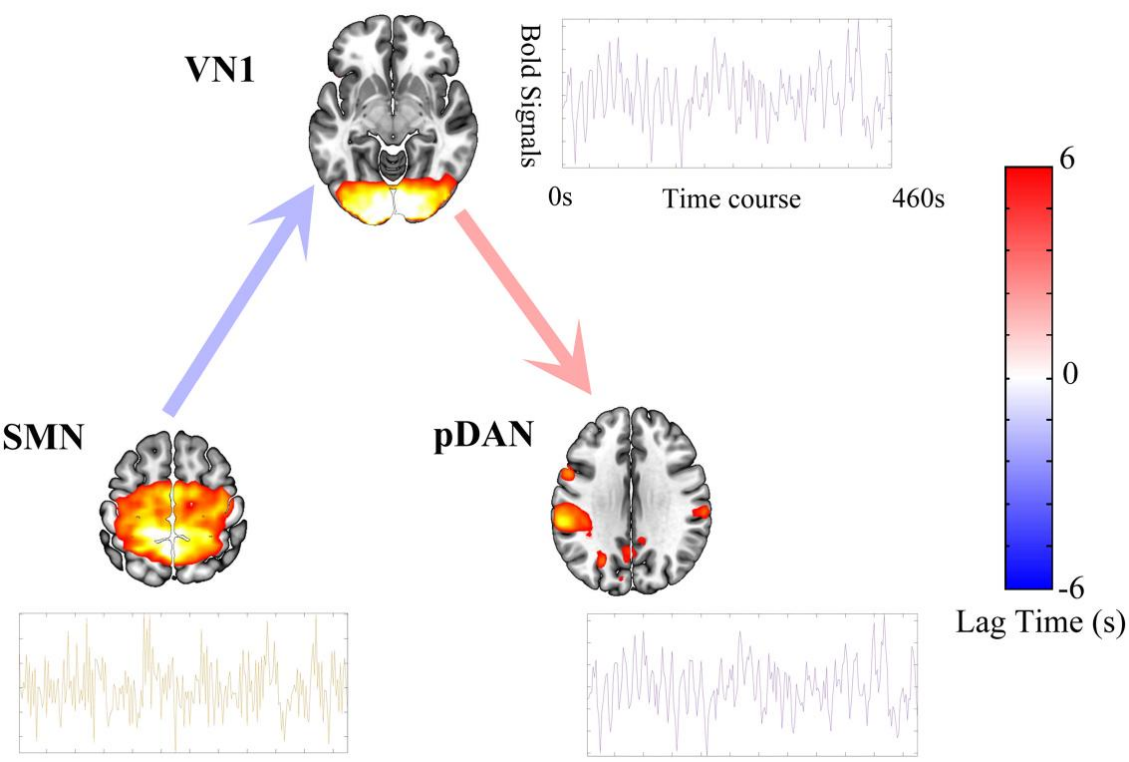
**Changed FNC following ccPAS intervention.** The colour of the arrows represents the value of Lag time. Cool colour indicates a shorter lag time, while warm colour indicates an extended lag time. SMN, sensorimotor network; VN1, primary VN; pDAN, posterior DAN (*N* = 15 subjects, Paired *t*-test *P* < 0.05, uncorrected).

The results of FNC analysis showed that before and after treatment in the ccPAS group, the time lag between VN1 and pDAN increased, while that between VN1 and SMN decreased (*P* < 0.05) (Fig. 6).

## Discussion

Our study demonstrates that ccPAS targeting the SMA and M1 induces large-scale reorganization of RSNs in chronic stroke patients, with distinct modulation patterns across motor, attentional and visual systems. The intervention selectively enhanced intra-network connectivity within the left FPN (LFPN), DAN and VN1, while reducing functional coupling in the right FPN (RFPN) and AN (Figs 2–5, Tables 1–4). Critically, the integration of the aDMN with sensorimotor regions (Table 2) and the shortened time lag between VN1 and SMN (Fig. 6) suggest that ccPAS can facilitate cross-modal plasticity and accelerates sensorimotor feedback—a sign of functional recovery.

These findings align with the network compensation hypothesis,^2^ where stroke disrupts distributed neural circuits, and recovery depends on compensatory rewiring of spared connections.^27^ This spontaneous process of repair is usually insufficient.^4^ By applying paired pulses with a +6 ms SMA→M1 interval, ccPAS likely potentiates cortico-cortical projections through STDP.^9^ The enhanced LFPN connectivity may reflect strengthened top-down control from prefrontal regions to motor planning areas, addressing the impaired ‘intention-to-action’ translation common in stroke.^28^ Conversely, the suppressed RFPN activity could indicate a rebalancing of interhemispheric competition, where reducing contralesional hyperactivity promotes functional reallocation to the affected hemisphere.^3^

The increased connectivity within VN1, pDAN and the strengthened coupling with SMN suggests that ccPAS induces a cross-modal plasticity that extends beyond the targeted motor network. Although the stimulation was applied at rest, we propose that enhancing the SMA-M1 pathway, a key node for sensorimotor integration, has upstream or downstream effects on interconnected networks. The visual cortex, particularly the dorsal stream, is densely connected to parietal and premotor areas and is critically involved in online visuomotor control and error monitoring.^18,29^ In the post-stroke brain, this visuomotor integration becomes more crucial because the patients often rely on visual feedback to compensate for impaired proprioception.^30^ Therefore, the ccPAS-induced facilitation of the SMA-M1 circuit may ‘strengthen’ the entire visuomotor pathway, leading to the observed strengthening of VNs and their functional coupling with the SMN. This reorganization likely represents a reinforcement of compensatory scaffolding mechanisms that would be engaged during subsequent motor tasks.

The functional integration of aDMN with sensorimotor regions strengthens its role in motor recovery. The aDMN may instead support self-referential processing during rehabilitation—enabling patients to evaluate consciously internal states (e.g. effort, fatigue) and adjust movement strategies.^31^ This integration could counteract maladaptive reliance on external cues, a common compensatory strategy in chronic stroke.^32^ The decreased pDMN connectivity with angular gyrus further suggests a shift away from default episodic memory retrieval towards goal-directed motor planning, consistent with task-fMRI studies of motor imagery.^8^

Our results drive a paradigm shift in neuromodulation; ccPAS not only stimulates local motor areas but also orchestrates network-level plasticity. The dual-target protocol—simultaneously stimulating SMA (motor initiation) and M1 (motor execution)—may ‘bridge’ disconnected cognitive motor networks, especially in patients with subcortical lesions sparing cortical hubs.^21^ For example, the enhanced DAN-SMN coupling could improve attention-driven motor control, benefiting patients with spatial neglect. Future trials should personalize stimulation sites based on lesion topology and integrate kinematic measures to validate network-behaviour correlations.

The observed effects likely arise from ccPAS-induced STDP at both local and distributed circuits. The +6 ms SMA→M1 interval potentiates feedforward projections from SMA (encoding movement goals) to M1 (executing commands),^23^ while global FC increases in DAN/LFPN suggest heterosynaptic plasticity through shared hubs. While this study provides novel insights into network-level plasticity induced by ccPAS, several limitations require attention. First, in terms of analytical methods, our study focused on functional connectivity between large-scale networks. To provide a more comprehensive theoretical basis of neuroplasticity across spatial scales, future analyses could be extended to include local measures of neural activity, such as the amplitude of low-frequency fluctuations and regional homogeneity. Furthermore, the use of directional modelling techniques like dynamic causal modelling in future studies could help identify the effective connectivity changes driven by ccPAS, while combining TMS-EEG could clarify the temporal dynamics of network modulation. Second, and most critically for clinical interpretation, our study lacks behavioural specificity. Although we observed reorganization in higher-order cognitive networks, there is an absence of dedicated behavioural measures for cognitive motor functions, which means that the functional implications of these changes remain speculative. The clinical scales employed in this study primarily reflect the overall motor function rather than isolating higher-order cognitive processes. Therefore, the proposed link between network changes and improved motor planning requires direct verification through task-based paradigms and specific cognitive assessments in future work.

In conclusion, this study is the first to provide evidence that ccPAS can selectively reconfigure RSNs in individuals with chronic stroke, bridging motor execution systems to higher-order cognitive networks. By demonstrating that non-invasive stimulation alters both intra- and inter-network dynamics, our work advances the therapeutic potential of network-targeted neuromodulation. These findings align with the network neurorehabilitation framework, in which recovery is driven by distributed plasticity rather than the recovery of isolated nodes.

## Data Availability

The datasets generated and/or analysed during the current study are available from the corresponding author on reasonable request. This study did not generate any custom code. All analyses were performed using the publicly available SPM12 software and the GIFT toolbox and FNC toolbox.
